# Prognostic Value of the Prognostic Nutritional Index in Patients with Metastatic Melanoma

**DOI:** 10.7150/jca.136818

**Published:** 2026-09-03

**Authors:** Mustafa Sahbazlar, Hikmet Akar, Atike Pinar Erdogan, Ferhat Ekinci

**Affiliations:** Department of Medical Oncology, Manisa Celal Bayar University Faculty of Medicine, Manisa, Turkey

**Keywords:** metastatic melanoma, prognostic nutritional index, systemic immune-inflammation index, neutrophil-to-lymphocyte ratio, survival, prognosis

## Abstract

**Background:**

Prognostic outcomes vary considerably among patients with metastatic melanoma. We examined the association of the prognostic nutritional index (PNI), neutrophil-to-lymphocyte ratio (NLR), and systemic immune-inflammation index (SII) with overall survival in this patient population.

**Methods:**

We retrospectively evaluated 60 patients with metastatic melanoma treated at a single center. PNI was derived from serum albumin and absolute lymphocyte count. A cut-off of 52 was identified by ROC analysis and used to define the PNI groups. Overall survival was analyzed using the Kaplan-Meier method and Cox proportional hazards regression. PNI was entered into separate multivariable models with NLR or SII.

**Results:**

Overall survival was longer in patients with PNI ≥52 than in those with PNI <52 (log-rank *p* = 0.008). PNI, NLR, SII, and immunotherapy use were associated with OS in univariate Cox analysis. After multivariable adjustment, PNI remained significant in the model containing NLR (HR: 0.36, 95% CI: 0.15-0.85; *p* = 0.020) and in the model containing SII (HR: 0.37, 95% CI: 0.16-0.88; *p* = 0.024). Immunotherapy use was also independently associated with OS, whereas NLR and SII were not. PNI remained associated with OS when analyzed as a continuous variable (HR: 0.91, 95% CI: 0.84-0.98; *p* = 0.008). The AUC for PNI was 0.706 (95% CI: 0.551-0.833; *p* = 0.015).

**Conclusion:**

Higher PNI was independently associated with longer overall survival in this cohort of patients with metastatic melanoma. These findings support further investigation of PNI as a readily available prognostic marker, although validation in larger independent cohorts is needed.

## Introduction

Malignant melanoma is an aggressive skin cancer with a considerable risk of metastatic spread. Although melanoma accounts for a relatively small proportion of skin cancers, it causes the majority of skin cancer-related deaths. Prognosis is strongly related to the extent of disease at diagnosis. Recent cancer statistics from the United States report a 5-year relative survival of approximately 95% across all stages, whereas this rate decreases to about 35% in patients with distant-stage disease 1.

Patients with metastatic melanoma have highly heterogeneous clinical outcomes despite recent therapeutic advances. Several clinicopathological, molecular, and laboratory factors, including ECOG performance status, serum lactate dehydrogenase (LDH), the presence of brain metastases, BRAF mutation status, and metastatic tumor burden, have been associated with prognosis 2,3. However, considerable variation in survival remains even among patients with similar clinical characteristics. This has encouraged the search for simple and readily available biomarkers that may provide additional prognostic information 4.

Malnutrition and systemic inflammation are common in patients with cancer and are associated with reduced treatment tolerance and poorer clinical outcomes 5,6. Serum albumin and peripheral lymphocyte count reflect different aspects of the host nutritional and immune status, and both have been associated with survival outcomes in patients with cancer 7. The Prognostic Nutritional Index (PNI) combines serum albumin and lymphocyte count and has shown prognostic value in several solid tumors 8-10.

Several blood-based inflammatory biomarkers, including the neutrophil-to-lymphocyte ratio (NLR) and systemic immune-inflammation index (SII), have also been investigated as prognostic markers in melanoma and other malignancies 11-13. Unlike NLR and SII, PNI also includes serum albumin and therefore incorporates a nutritional component into the assessment.

Evidence for PNI in advanced melanoma, however, remains limited. Previous studies have reported associations between PNI and clinical outcomes, but data on its independent prognostic value for survival are still scarce 14-16. In the present study, we evaluated the prognostic significance of PNI in patients with metastatic melanoma and compared its prognostic performance with NLR and SII.

## Materials and Methods

### Study Design and Patient Selection

This retrospective, single-center cohort study included patients diagnosed with malignant melanoma and followed at the Department of Medical Oncology, Manisa Celal Bayar University Faculty of Medicine, between 2010 and 2021.

Patients who developed metastatic disease (stage IV) during the course of their illness were included. Accordingly, both patients presenting with de novo metastatic disease and those who developed metastasis during follow-up after an initial diagnosis of earlier-stage disease (metachronous metastasis) were evaluated. Stage refers to disease stage at initial diagnosis, and all patients were analyzed at the time of metastatic disease.

Patients with histopathologically confirmed malignant melanoma who had available serum albumin and absolute lymphocyte count measurements during the metastatic period (at diagnosis or prior to systemic therapy), and whose clinical follow-up data were complete, were included. Patients with active infection, chronic inflammatory disease, concomitant hematological disorders, or missing essential clinical or laboratory data were excluded.

The study protocol was approved by the Manisa Celal Bayar University Faculty of Medicine Health Sciences Ethics Committee (approval number: 20.478.486, date: 09 March 2022). Due to the retrospective design of the study and the use of anonymized data, the requirement for written informed consent was waived by the ethics committee in accordance with national regulations and the Declaration of Helsinki. All patient data were anonymized prior to analysis.

### Data Collection

Demographic and clinical data (age, sex, smoking history, ECOG performance status, tumor thickness, ulceration status, TNM stage, melanoma subtype, sites of metastasis, BRAF mutation status, and administered treatments) were obtained from the hospital electronic medical records system.

Tumor staging was performed according to the 8th edition of the AJCC staging system.

Data for some variables were not available for all patients. Data required for the calculation of PNI were missing in 3 patients; therefore, these patients were excluded from PNI-related analyses. T category, ulceration, and Breslow thickness data were available for 55 patients, while nodal status was evaluated in 59 patients. BRAF mutation analysis was performed in 36 patients. Therefore, the number of patients included in each analysis varied depending on the variable evaluated.

### Calculation of Prognostic Nutritional Index (PNI)

PNI was calculated according to the formula originally described by Onodera et al. 17: PNI = 10 × albumin [g/dL] + 0.005 × absolute lymphocyte count [/mm³]. The absolute lymphocyte count was expressed as cells/mm³.

The systemic immune-inflammation index (SII) was calculated as platelet count × neutrophil count / lymphocyte count using absolute peripheral blood counts. SII was expressed as ×10⁴ for ease of interpretation and modeled per ×10⁴ increase in regression analyses.

The neutrophil-to-lymphocyte ratio (NLR) was calculated by dividing the absolute neutrophil count by the absolute lymphocyte count.

Laboratory values obtained at the time of metastatic disease were used for PNI calculation. Patients were stratified into low and high PNI groups based on a cut-off value of 52 determined by ROC analysis.

### Endpoints

Overall survival (OS) was the primary endpoint and was calculated from the date of metastatic disease diagnosis to death from any cause.

### Statistical Analysis

All analyses were conducted using IBM SPSS Statistics for Windows, version 31.0 (IBM Corp., Armonk, NY, USA). The distribution of continuous variables was examined with the Shapiro-Wilk test. Continuous data are reported as mean ± standard deviation or median (minimum-maximum), according to their distribution.

Student's t-test or the Mann-Whitney U test was used to compare continuous variables between two groups, as appropriate. Categorical variables are presented as numbers and percentages and were compared with the chi-square or Fisher's exact test. Fisher's exact test was preferred when expected cell counts were <5. Cohen's d was calculated as an effect-size measure for continuous variables.

Overall survival was estimated by the Kaplan-Meier method, and survival curves were compared with the log-rank test. Associations with OS were examined using Cox proportional hazards regression. Variables with p < 0.10 in univariate analysis were included in the multivariable analysis. Because PNI, NLR, and SII contain related hematological components, two separate multivariable models were fitted: one containing PNI and NLR and the other containing PNI and SII. Immunotherapy use was included in both models. Hazard ratios (HRs) are reported with 95% confidence intervals (CIs).

PNI was also entered into a Cox model as a continuous variable to determine whether its association with OS was dependent on the ROC-derived cut-off. Analyses were performed on available cases; patients with missing data for a given variable were excluded from analyses involving that variable. Statistical significance was defined as a two-sided *p* < 0.05. No formal sample size calculation was performed because of the retrospective study design.

## Results

The study included 60 patients with a mean age of 62.0 ± 11.9 years; 32 (53.3%) were male. ECOG performance status was 0-1 in 88.3% of patients, and 85.0% had undergone surgery. Nodal involvement was observed in 89.8% of evaluable patients. Stage IV disease at initial diagnosis was recorded in 71.7% of the cohort. Regarding melanoma subtype, 38 patients (63.3%) had cutaneous melanoma, 12 (20.0%) had acral melanoma, 5 (8.3%) had ocular melanoma, and 5 (8.3%) had mucosal melanoma. No patient had melanoma of unknown primary. Baseline characteristics are presented in Table 1.

Using a PNI cut-off of 52, patients were divided into low- and high-PNI groups. Mean age was 63.9 ± 11.5 years in the PNI <52 group and 57.8 ± 12.1 years in the PNI ≥52 group; the difference did not reach statistical significance (*p* = 0.066). No significant differences between the groups were observed for sex, ECOG performance status, de novo versus metachronous metastatic disease, or liver metastasis. Ulceration was more common in patients with PNI ≥52 (78.9% vs. 41.2%, *p* = 0.008). NLR was higher in the PNI <52 group (3.28 ± 1.59 vs. 2.13 ± 0.94, *p* = 0.004; Cohen's d = 0.82). Similarly, SII levels were significantly higher in the low PNI group (80.4 [25.3-202.0] vs. 51.9 [17.2-95.4], *p* = 0.004). In contrast, immunotherapy use in the metastatic setting did not differ significantly between the groups (51.4% vs. 45.0%, *p* = 0.647). These findings are presented in Table 2.

First-line systemic treatments are summarized in Supplementary Table S1. During the metastatic disease course, 12 patients (20.0%) received anti-PD-1 monotherapy, 17 (28.3%) received anti-CTLA-4 monotherapy, and 9 (15.0%) received BRAF/MEK inhibitors. No patient received combination immunotherapy. The distribution of systemic treatment classes administered during metastatic disease is shown in Supplementary Table S2.

**Figure 1:**
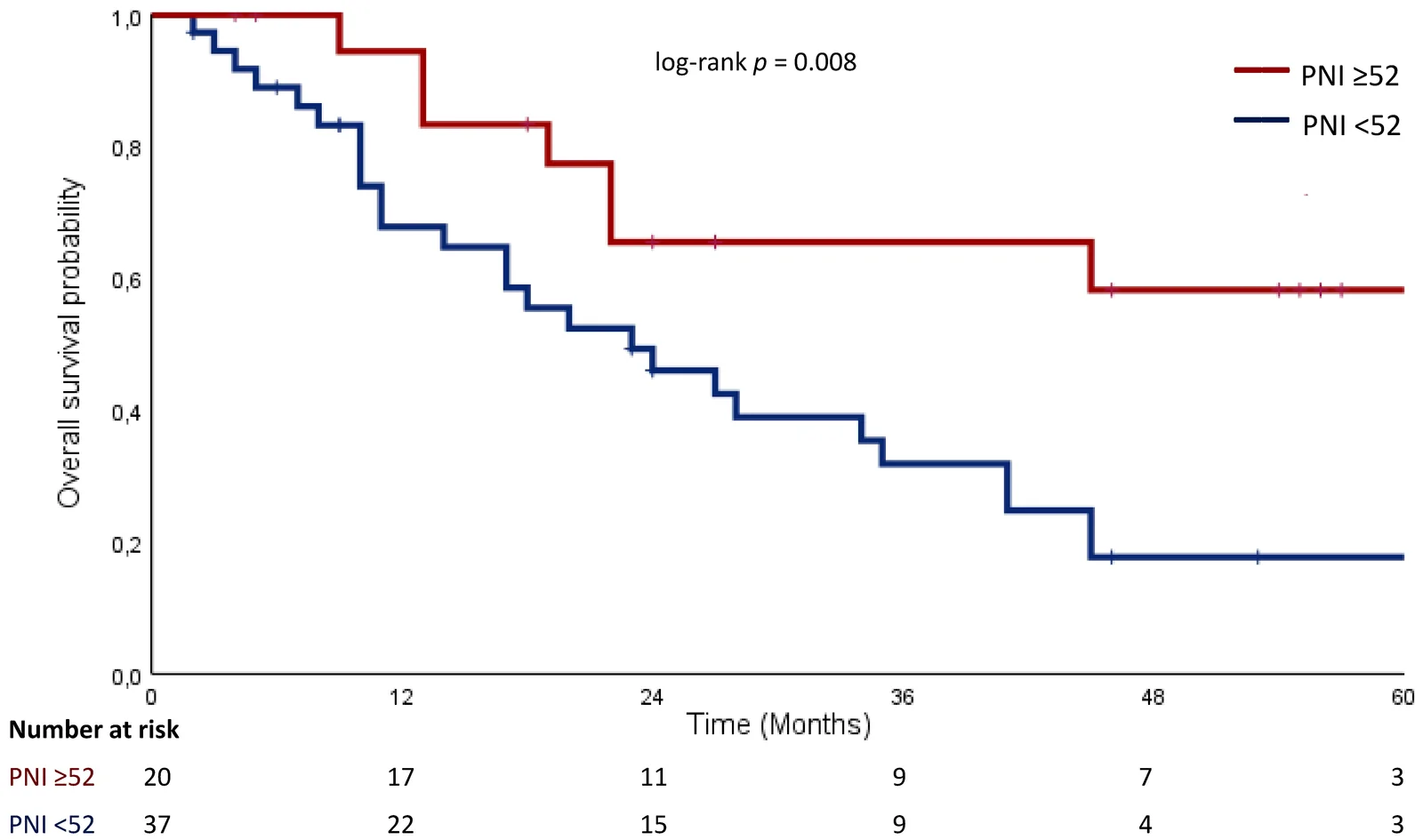
Kaplan-Meier curves for overall survival according to PNI (<52 vs ≥52). Tick marks indicate censored observations. Numbers at risk are shown below the x-axis.

The median follow-up duration was 21.0 months (range, 1.5-107.4). Kaplan-Meier analysis showed that patients with PNI ≥52 had significantly longer overall survival compared to those with PNI < 52 (log-rank *p* = 0.008) (Figure 1).

In univariate Cox regression analysis, PNI (HR: 0.35, 95% CI: 0.15-0.81, *p* = 0.014), NLR (HR: 1.25, 95% CI: 1.01-1.55, *p* = 0.039), SII (HR: 1.01, 95% CI: 1.00-1.02, *p* = 0.004), and immunotherapy use (HR: 0.42, 95% CI: 0.21-0.83, *p* = 0.013) were significantly associated with overall survival. Other clinical variables, including sex, ECOG performance status, T category, nodal involvement, ulceration, and melanoma subtype, were not significantly associated with survival (Table 3).

In the multivariate model including PNI, NLR, and immunotherapy, PNI (HR: 0.36, 95% CI: 0.15-0.85, *p* = 0.020) and immunotherapy use (HR: 0.42, 95% CI: 0.20-0.84, *p* = 0.015) remained independent prognostic factors for overall survival, whereas NLR was no longer significant (*p* = 0.338) (Table 4).

In an alternative multivariate model including PNI, SII, and immunotherapy, PNI (HR: 0.37, 95% CI: 0.16-0.88, *p* = 0.024) and immunotherapy use (HR: 0.43, 95% CI: 0.21-0.87, *p* = 0.019) remained independent prognostic factors for overall survival, while SII was not significantly associated with survival (*p* = 0.150) (Table 4).

When analyzed as a continuous variable, PNI also remained significantly associated with overall survival (HR: 0.91, 95% CI: 0.84-0.98, *p* = 0.008), indicating that increasing PNI values were associated with a reduced risk of mortality.

**Figure 2:**
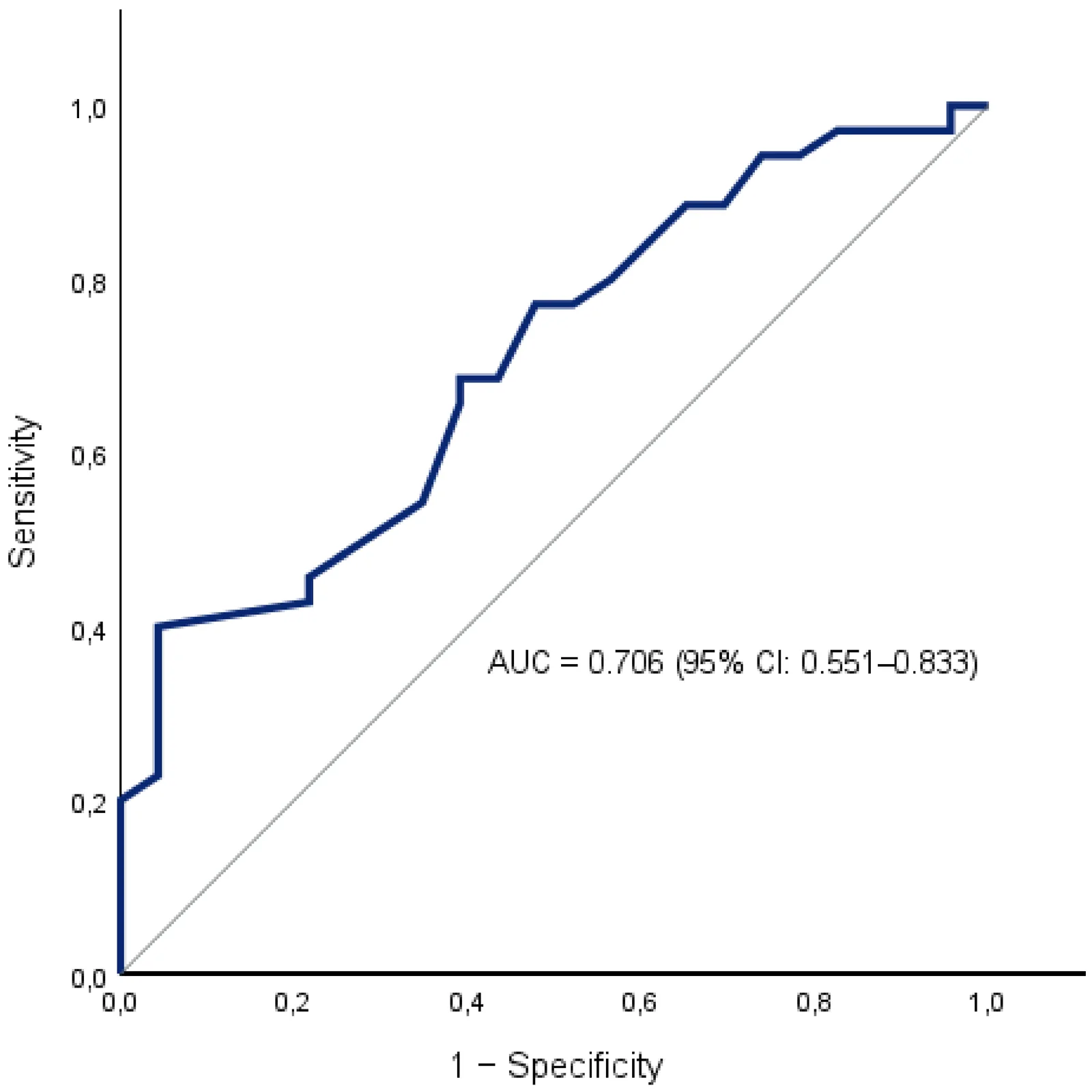
Receiver operating characteristic (ROC) curve of PNI for predicting overall survival. The area under the curve (AUC) was 0.706 (95% CI: 0.551-0.833). The diagonal line represents the line of no discrimination.

The AUC for PNI was 0.706 (95% CI: 0.551-0.833; *p* = 0.015). At a PNI cut-off of 52, sensitivity was 79.4% and specificity was 56.5% (Figure 2).

## Discussion

The main finding of this study was the association between PNI and overall survival in patients with metastatic melanoma. Patients with PNI ≥52 had significantly longer survival, and PNI remained independently associated with OS after adjustment for other prognostic variables. PNI remained significant in both multivariable models, whether NLR or SII was included. In contrast, NLR and SII were associated with survival in univariate analyses but did not remain significant after adjustment.

Only a limited number of studies have specifically examined PNI in melanoma. Mirili et al. reported a pattern similar to ours: NLR, SII, and PNI were associated with OS in univariate analysis, whereas PNI remained independently associated with OS after multivariable adjustment and NLR and SII did not 14. Hannarici et al. similarly found longer PFS and OS in patients with higher PNI, although PNI did not retain independent significance in their multivariable models 15. More recently, Deng et al. evaluated inflammatory and nutritional markers during PD-1-based therapy in advanced melanoma. PNI was higher and remained relatively stable among patients who achieved clinical benefit, although it was not retained in their final multivariable predictive model 16. Taken together, the available melanoma studies suggest that PNI is related to outcome, although its independent prognostic role is less clear. In our cohort, which included only patients with metastatic disease, baseline PNI remained independently associated with OS.

The prognostic value of inflammatory indices has been more extensively studied in melanoma. Previous meta-analyses have generally associated elevated NLR and other systemic inflammatory markers with poorer outcomes, including among patients receiving immune checkpoint inhibitors 11,12. SII has also been associated with OS and PFS in patients treated with immune checkpoint inhibitors across different malignancies 13. In our cohort, both NLR and SII were associated with OS in univariate analysis, but neither retained statistical significance in the multivariable models. This may partly reflect the biological overlap between these indices, which are calculated from related peripheral blood cell counts. PNI differs from these markers because it also incorporates serum albumin. Whether this nutritional component accounts for the stronger association between PNI and survival observed in our cohort cannot be determined from the present data.

The PNI cut-off identified in our cohort was 52. Previous melanoma studies have reported different thresholds, including approximately 40 in the study by Mirili et al. and 51.5 in the study by Hannarici et al. 14,15. Such variation may reflect differences in patient populations, disease stage, treatment exposure, and methods used to determine the optimal threshold. Importantly, PNI was also associated with survival when analyzed as a continuous variable. Thus, the survival association was not limited to the ROC-derived threshold of 52.

The discriminatory performance of PNI in ROC analysis was moderate. Therefore, PNI should not be considered a stand-alone tool for individual risk prediction based on the present results. Its potential value lies in its simplicity and availability, since both albumin and lymphocyte count are routinely measured in clinical practice. The relatively high sensitivity at the selected cut-off is of interest, but this threshold was derived from the same cohort in which its prognostic performance was evaluated. For this reason, the cut-off of 52 requires validation in an independent cohort before it can be considered for clinical use.

Immunotherapy use was also independently associated with improved survival in our analysis. This finding should be interpreted cautiously because treatment allocation was not randomized. Because this was a retrospective cohort, we cannot determine how much of the observed survival difference was attributable to immunotherapy itself. Treatment selection may have been influenced by performance status, disease characteristics, treatment era, access to therapy, and other factors not captured in our dataset. This is particularly important because the study period covered substantial changes in the systemic treatment of metastatic melanoma.

Several limitations should be considered. This was a retrospective, single-center study with a relatively small sample, and the limited number of events restricted the number of variables that could reasonably be included in the multivariable models. Model stability is therefore a concern. We used separate models for PNI with NLR and SII to reduce overlap between these indices, although residual collinearity cannot be excluded. Another limitation is that the PNI cut-off was derived from the same cohort and was not validated. BRAF status was unavailable in a substantial proportion of patients, limiting adjustment for tumor biology. Finally, our analyses were based on baseline inflammatory and nutritional measurements. We did not examine their changes during treatment. This may be relevant, as Deng et al. recently reported that longitudinal changes in these parameters were associated with treatment outcome 16.

The simultaneous evaluation of PNI, NLR, and SII in the same metastatic melanoma cohort allowed direct comparison of their associations with survival. We also examined PNI both at the ROC-derived cut-off and as a continuous variable. These are useful features of the analysis, but they do not overcome the limitations of the retrospective design and small sample size. The results therefore need confirmation in larger independent cohorts.

In this metastatic melanoma cohort, PNI was independently associated with overall survival, whereas NLR and SII did not remain significant after multivariable adjustment. PNI is readily available from routine laboratory measurements, but its prognostic role and the cut-off identified here need validation in larger independent cohorts before clinical use can be considered.

## Supplementary Material

Supplementary tables.

## AI Statement

The authors used ChatGPT (OpenAI) solely for English language editing. All scientific content, data analysis, interpretation, and conclusions were developed and approved by the authors.

## Figures and Tables

**Table 1:** Baseline demographic and clinical characteristics of the study population (n=60).

Variable	n (%)
Age, years, mean ± SD	62.0 ± 11.9
Sex	
Male	32 (53.3)
Female	28 (46.7)
Smoking status (n=59)	
Current smoker	9 (15.3)
Former smoker	10 (16.9)
Never smoker	40 (67.8)
ECOG performance status	
0-1	53 (88.3)
≥2	7 (11.7)
Ulceration (Yes)	31 (56.4)
Breslow thickness, mm (n=55)	
<1 mm	4 (7.3)
1-2 mm	7 (12.7)
2-4 mm	19 (34.5)
>4 mm	25 (45.5)
Nodal involvement (N+)	53 (89.8)
Metastatic presentation	
De novo metastatic	43 (71.7)
Metachronous metastatic	17 (28.3)
Melanoma subtype	
Cutaneous	38 (63.3)
Acral	12 (20.0)
Ocular	5 (8.3)
Mucosal	5 (8.3)
BRAF mutation (n=36)	
Mutant	14 (38.9)
Wild-type	22 (61.1)
Sites of metastasis	
Liver	14 (23.3)
Lung	34 (56.7)
Bone	16 (26.7)
Brain	14 (23.3)
Immunotherapy (Yes)	28 (46.7)

ECOG: Eastern Cooperative Oncology Group performance status.Data were missing for some variables: Breslow thickness (n=5), nodal involvement (n=1), ulceration (n=5), and smoking (n=1). BRAF mutation data were available for 36 patients (60% of the cohort).

**Table 2:** Comparison of clinical and laboratory characteristics according to PNI groups (n=57)

Variable	PNI <52 (n = 37)	PNI ≥52 (n = 20)	*p*-value
Age, years, mean ± SD	63.9 ± 11.5	57.8 ± 12.1	0.066
Male, n (%)	21 (56.8)	9 (45.0)	0.396
Female, n (%)	16 (43.2)	11 (55.0)	
ECOG 0-1, n (%)	32 (86.5)	18 (90.0)	1.000
ECOG ≥2, n (%)	5 (13.5)	2 (10.0)	
De novo metastatic, n (%)	27 (73.0)	14 (70.0)	0.812
Metachronous metastatic, n (%)	10 (27.0)	6 (30.0)	
Ulceration (Yes), n (%)	14 (41.2)	15 (78.9)	0.008
Liver metastasis (Yes), n (%)	7 (18.9)	5 (25.0)	0.736
NLR, mean ± SD	3.28 ± 1.59	2.13 ± 0.94	0.004
SII (×10⁴), median (range)	80.4 (25.3-202.0)	51.9 (17.2-95.4)	0.004
Immunotherapy (Yes), n (%)	19 (51.4)	9 (45.0)	0.647

Abbreviations: PNI, prognostic nutritional index; ECOG, Eastern Cooperative Oncology Group performance status; NLR, neutrophil-to-lymphocyte ratio; SII, systemic immune-inflammation index; SD, standard deviation. Ulceration percentages were calculated among patients with available ulceration data (PNI <52, n=34; PNI ≥52, n=19).

**Table 3:** Univariate Cox regression analysis of factors associated with OS

Variable	HR (95% CI)	*p*-value
PNI (≥52 vs <52)	0.35 (0.15-0.81)	0.014
NLR	1.25 (1.01-1.55)	0.039
SII (per 10⁴ increase)	1.01 (1.00-1.02)	0.004
Immunotherapy (Yes vs. No)	0.42 (0.21-0.83)	0.013
Sex (Male vs. Female)	0.87 (0.45-1.66)	0.665
ECOG (≥2 vs. 0-1)	1.32 (0.54-3.20)	0.542
T category (T3-4 vs. T1-2)	1.29 (0.53-3.11)	0.578
Nodal involvement (N+ vs. N0)	1.03 (0.31-3.43)	0.966
Ulceration (Yes vs. No)	0.75 (0.38-1.48)	0.408
Melanoma subtype	Not estimable*	

Abbreviations: HR, hazard ratio; CI, confidence interval; PNI, prognostic nutritional index; NLR, neutrophil-to-lymphocyte ratio; SII, systemic immune-inflammation index; ECOG, Eastern Cooperative Oncology Group performance status. *HR could not be estimated due to sparse data and lack of events. HR values represent comparisons relative to the reference category.

**Table 4:** Multivariate Cox regression analysis of independent prognostic factors

Variables	Model 1 HR (95% CI)	*p*-value	Model 2 HR (95% CI)	*p*-value
PNI (≥52 vs. <52)	0.36 (0.15-0.85)	0.020	0.37 (0.16-0.88)	0.024
NLR	1.11 (0.89-1.39)	0.338	—	—
SII (per 10⁴ increase)	—	—	1.01 (1.00-1.02)	0.150
Immunotherapy (Yes vs. No)	0.42 (0.20-0.84)	0.015	0.43 (0.21-0.87)	0.019

Abbreviations: HR, hazard ratio; CI, confidence interval; PNI, prognostic nutritional index; NLR, neutrophil-to-lymphocyte ratio; SII, systemic immune-inflammation index. Model 1: PNI + NLR + Immunotherapy Model 2: PNI + SII + Immunotherapy

## Data Availability

The datasets used and/or analyzed during the current study are available from the corresponding author on reasonable request.
