# Supplementary material for: Prognostic Value of the Prognostic Nutritional Index in Patients with Metastatic Melanoma
Source: J Cancer. 2026 Sep 3;17(9):1576–82. doi: 10.7150/jca.136818 (PMC13573299; doi:10.7150/jca.136818)
Supplement: Supplementary file 1 [file jcav17p1576s1.pdf]

Table S1. First-line systemic treatment administered for metastatic melanoma (n=60)

First-line systemic treatment	n (%)
No systemic treatment	11 (18.3)
Ipilimumab	8 (13.3)
Dabrafenib + Trametinib	4 (6.7)
Temozolomide	25 (41.7)
Carboplatin + Paclitaxel	9 (15.0)
Vemurafenib + Cobimetinib	3 (5.0)

Eleven patients (18.3%) did not receive systemic treatment because of poor performance status, rapid clinical deterioration, patient preference, or other clinical considerations.

Table S2. Systemic therapies administered during the metastatic disease course (n = 60)

Systemic therapy	n (%)
Anti-PD-1 monotherapy (nivolumab)	12 (20.0)
Anti-CTLA-4 monotherapy (ipilimumab)	17 (28.3)
Combination immunotherapy	0 (0.0)
BRAF/MEK inhibitors	9 (15.0)
Temozolomide	34 (56.7)
Taxane-containing chemotherapy	18 (30.0)
Platinum-containing chemotherapy	15 (25.0)

Patients may have received more than one treatment class during the metastatic disease course; therefore, treatment categories are not mutually exclusive and percentages do not sum to 100%. BRAF/MEK inhibitors included dabrafenib–trametinib and vemurafenib–cobimetinib.
